# Affective and psychotic reactivity to daily-life stress in adults with 22q11DS: a study using the experience sampling method

**DOI:** 10.1186/s11689-020-09333-2

**Published:** 2020-11-13

**Authors:** Maude Schneider, Thomas Vaessen, Esther D. A. van Duin, Zuzana Kasanova, Wolfgang Viechtbauer, Ulrich Reininghaus, Claudia Vingerhoets, Jan Booij, Ann Swillen, Jacob A. S. Vorstman, Thérèse van Amelsvoort, Inez Myin-Germeys

**Affiliations:** 1 Clinical Psychology Unit for Intellectual and Developmental Disabilities, Faculty of Psychology and Educational Sciences, University of Geneva, Boulevard du Pont d’Arve 40, 1205 Geneva, Switzerland; 2 Center for Contextual Psychiatry, Department of Neurosciences, KU Leuven, Kapucijnenvoer 33 Bus 7001 (Blok H), 3000 Leuven, Belgium; 3 Department of Psychiatry & Neuropsychology, Maastricht University, Minderbroedersberg 4-6, Maastricht, 6211 LK The Netherlands; 4 Institute for Interdisciplinary Studies, University of Amsterdam, PO Box 94224, Science Park 904, Amsterdam, 1090 GE The Netherlands; 5 Centre for Epidemiology and Public Health, Health Service and Population Research Department, Institute of Psychiatry, Psychology & Neuroscience, David Goldberg Centre, King’s College London, 18 De Crespigny Park, London, SE5 8AF UK; 6 Department of Public Mental Health, Central Institute of Mental Health, Medical Faculty Mannheim, Heidelberg University, Square J5, 68159 Mannheim, Germany; 7 Department of Radiology and Nuclear Medicine, Amsterdam University Medical Centers, University of Amsterdam, Meibergdreef 9, Amsterdam, 1105 AZ The Netherlands; 8 Department of Human Genetics, KU Leuven, Herestraat 49, 3000 Leuven, Belgium; 9 Center for Human Genetics, Hospital Gasthuisberg, Herestraat 49, 3000 Leuven, Belgium; 10 Department of Psychiatry, The Hospital for Sick Children and University of Toronto, 555 University Avenue, Burton Wing, Toronto, Ontario M5G 1X8 Canada; 11 Program in Genetics and Genome Biology, Research Institute, The Hospital for Sick Children, 686 Bay St., Toronto, Ontario M5G 0A4 Canada

**Keywords:** Experience sampling method, 22q11.2 deletion syndrome, Stress reactivity, Positive affect, Negative affect, Momentary psychotic experiences

## Abstract

**Background:**

22q11.2 deletion syndrome (22q11DS) is a genetic disorder associated with an increased risk of psychiatric disorders. Vulnerability for psychopathology has been related to an increased reactivity to stress. Here, we examined affective states, perceived stress, affective and psychotic reactivity to various sources of environmental stress using the experience sampling method (ESM), a structured diary technique allowing repeated assessments in the context of daily life.

**Methods:**

Adults with 22q11DS (*n* = 31; age, 34.1 years) and matched healthy controls (HCs; *n* = 24; age, 39.9 years) were included. ESM was used to assess affective states, perceived stress, and stress reactivity. Data were analyzed using multilevel regression models.

**Results:**

Adults with 22q11DS displayed overall higher levels of negative affect but comparable levels of positive affect compared to HCs. Higher levels of perceived stress were reported by individuals with 22q11DS. Comparable affective and psychotic reactivity in relation to all types of environmental stress was observed between the two groups.

**Conclusion:**

The results point toward higher levels of negative affect and differences in the perception of daily hassles in 22q11DS but no difference in affective or psychotic reactivity to stress. This study contributes to the growing literature regarding the impact of stress on the development of psychopathology in the 22q11DS population.

## Introduction

The 22q11.2 deletion syndrome (22q11DS) is one of the most common recurrent copy number variant disorders occurring in approximately 1 in 2000‑4000 births and is caused by a microdeletion resulting in hemizygosity for approximately 50 genes [1]. 22q11DS is associated with a variety of symptoms including physical, social, cognitive, and psychiatric problems. Besides a high prevalence of neurodevelopmental disorders (25‑50%), anxiety and mood disorders are reported in 15‑65% of the individuals with 22q11DS, and the syndrome is characterized by high rates (20‑30%) of psychotic disorders [2, 3].

The relation between stress and psychopathology is likely bidirectional. Partially as a result of the cognitive, mental, social, and physical challenges associated with the syndrome, individuals with 22q11DS are thought to experience increased (chronic) stress and anxiety, often already present from childhood onwards [4]. Abnormal levels of (chronic) experienced (early life) stress have, in turn, been associated to increased risk for a wide range of psychiatric disorders (e.g., major depression, psychotic disorders, anxiety disorders, and posttraumatic stress disorder) [5–8], especially in vulnerable individuals [9, 10]. This is thought to be caused by sensitization or dysregulation of the hypothalamic-pituitary-adrenocortical (HPA) axis [11], responsible for the bodily stress response characterized by the secretion of, among others, the hormone cortisol [12]. A dysfunctional HPA axis could lead to abnormal stress reactivity, which is defined as the affective response to stressful events [13–15].

It has been proposed that psychiatric disorders, including psychosis, emerge in vulnerable individuals under the influence of environmental stressors, through a process of stress sensitization [16]. Stress sensitization is conceptualized as an increase in the response to an environmental stressor with repeated exposure to this stressor, eventually leading to long-lasting changes [16]. Vulnerability to stress is reflected in affective sensitivity, which can be observed in response to daily environmental stressors [17–19]. Increased stress reactivity has been shown in individuals with psychotic disorders [19, 20] as well as in their siblings [21], suggesting an heritable component to stress reactivity. Interestingly, increased stress reactivity has also been linked to the persistence of psychotic experiences in the general population [13], indicating that stress sensitivity might be a mechanism underlying both the emergence and persistence of psychotic experiences, which in turn is a significant predictor for the development of a psychotic disorder [22]. Evidence for a role of stress in the development of psychiatric disorders in 22q11DS comes from a recent study showing a significant association between increased exposure to stressful life events in the preceding year and higher levels of subthreshold psychotic symptoms [23]. However, this study does not address the issue of affective sensitivity to stressful events that happen regularly in the flow of daily life (e.g., missing a bus or feeling uncomfortable in a social situation).

The experience sampling method (ESM) is found to be a reliable method to assess affective reactivity to daily-life stress in vulnerable populations [24]. ESM is a structured diary technique that collects data in the flow of daily life through multiple assessments taking place during several days. However, no study to date has used ESM in 22q11DS to investigate affective reactivity to daily-life stress. Here, we used this methodology for the first time in a sample of adults with 22q11DS to investigate affect, stress, and affective reactivity to various types of daily environmental stressors (related to the social sphere, the activity that the person is doing, or the events that occurred), allowing for a reliable assessment of the interaction between personal vulnerability and environmental stressors in real life. We closely examined reactivity to social stressors, given that 22q11DS is characterized by decreased social skills [25], high rates of social anxiety [3], high levels of maladaptive social behaviors [25], and an atypical processing of social information (e.g., [26]), which may lead to more negative appraisals of social interactions in daily life.

In light of the increased rates of psychopathology in this population [3], we hypothesized that compared to healthy controls (HCs), adults with 22q11DS would show higher levels of negative affect, higher levels of perceived stress in different contexts (i.e., related to the social sphere, the current activity, and the events that occurred in daily life) and altered affective and psychotic reactivity to stress.

## Methods

### Sample

In total, 31 individuals with 22q11DS were recruited through the Dutch 22q11DS family network, the National Adult 22q11DS Outpatient Clinic at Maastricht University Medical Center, the National Children 22q11DS Outpatient Clinic at University Medical Center Utrecht in the Netherlands, and the University Hospital Leuven in Belgium. In addition, individuals with 22q11DS that participated in previous studies were also contacted. The results obtained in the 22q11DS sample were compared to those obtained in a sample of 24 HCs. Recruitment and inclusion criteria for the 22q11DS and HC subjects are as described previously [27, 28]. The participants included in the present study are overlapping with those included in van Duin et al. [28].

This study was approved by the Medical Ethical Committee of the University of Maastricht (The Netherlands) according to the standard of the National Committee of Health Research Ethics. Written informed consent was obtained from all participants included in the study.

Inclusion criteria for all participants were (1) age between 18 and 60 years, (2) sufficient command of the Dutch language, (3) mental competence (for the 22q11DS group, this was confirmed by a psychiatrist during an interview before inclusion in the study), and (4) additionally for 22q11DS subjects, there had to be a confirmed deletion at chromosome 22q11DS (determined by fluorescence in situ hybridization (FISH), multiplex ligation-dependent probe amplification (MLPA), or micro-array analysis) [29]. General exclusion criteria for all participants were (1) current severe endocrine, cardiovascular or neurological disease, (2) current alcohol and/or cannabis dependence (confirmed by the substance abuse module of the Composite International Diagnostic Interview (CIDI; [30]). Additionally, exclusion criteria for the HC group only were (3) having a lifetime history of axis I or II disorders as determined by the mini-international neuropsychiatric interview (M.I.N.I; [31]). and (4) current use of neuroleptics, steroids, or thyroid medication.

### General procedure

The current study was carried out during two meetings (either in the living situation of the participant or at the university department). During the first meeting, participants completed different behavioral questionnaires and were trained and briefed about the ESM procedure with the PsyMate^TM^ (www.psymate.eu; [32]), an electronic device used for within-day self-assessment. The ESM protocol was carried out by the participants between the first and the second meeting, with a few telephone calls from the researchers to support and verify study compliance. During the second meeting, the PsyMate^TM^ device was recollected and the participants were debriefed about the independent ESM period.

### Questionnaires/behavioral assessments

Information about demographic characteristics and medication use were collected during the briefing session (i.e., before the start of the ESM data collection week). Presence of current mental disorders was assessed using the M.I.N.I. [31]. An estimated full-scale IQ was assessed with the shortened Wechsler Adult Intelligence Scale (WAIS-II-NL; [33, 34]). For additional information regarding the behavioral assessments in the HC group, see Kasanova et al. [27].

### Assessment in daily life (ESM protocol)

ESM is a data collection diary method in which participants self-evaluate their experiences in a natural setting throughout their daily life [24]. Previous studies using ESM in psychiatric patients have demonstrated the feasibility, validity, and reliability of this method in vulnerable populations (e.g., [20, 35]). To collect information in daily life, participants received the PsyMate^TM^ [32], an electronic dedicated device with a touch screen. This device was programmed to beep at 10 semi-random times per day on 6 consecutive days between 7:30 h and 22:30 h. Participants were instructed to fill out a short questionnaire on the Psymate^TM^ after each beep. They were familiarized with the device during the briefing session and performed a test run of the ESM questionnaire during which the meaning of all the items was explained. In line with previous studies (e.g., [20, 35]), only participants who provided full responses to at least one-third of the beeps in total were kept in the analyses and incomplete sample moments were excluded.

Positive affect (PA), negative affect (NA), and momentary psychotic experiences were assessed at each beep using a series of items (Table 1). Participants then had to report whether or not they were in company of other persons (social context). Based on this answer, the social context was divided in three categories: (1) alone; (2) with familiar persons (i.e., colleagues, family, friends, flatmates, or partner); or (3) with strangers. If participants reported to be alone, they were asked to report about their current appraisal of aloneness (alone stress; Table 1). If they reported to be with familiar persons or strangers, they were asked to report about their current appraisal of this social company (social stress; Table 1). Participants were also asked to report about their current activity (school/work-related activities; house-related activities (grocery/household chores, activities related to self-care); social activities (social contact, online social contact, taking care of other people); leisure activities (active leisure activity, passive leisure activity, sport); eating/drinking; something else) and to appraise the stress related to this activity (activity-related stress; Table 1). Finally, they were asked to think about the most important event that happened since the last beep and to rate the pleasantness of this event (event-related stress; Table 1).

**Table 1:** ESM questions used to compute variables for different domains

Domain	Aggregate ESM measure
**Negative affect**	Negative affect was based on the average score of 5 items. “I feel irritated,” “I feel anxious,” “I feel insecure,” “I feel guilty,” “I feel down.” These items were rated on a 7-point Likert scale ranging from 1 to 7. Mean score of the 5 items was taken to compute the negative affect value, with higher scores representing higher negative affect.
**Positive affect**	Negative affect was based on the average score of 3 items. “I feel Cheerful,” “I feel relaxed,” “I feel enthusiastic.” These items were rated on a 7-point Likert scale ranging from 1 to 7. Mean score of the 3 items was taken to compute the positive affect value, with higher scores representing higher positive affect.
**Psychotic experiences**	Momentary psychotic experiences were based on the average score of items. “I feel unreal” and “I feel suspicious.” These items were rated on a 7-point Likert scale ranging from 1 to 7. Mean score of the 2 items was taken to compute the momentary psychotic experiences value, with higher scores representing higher psychotic experiences.
**Social stress**	Social stress was based on the appraisal of the current social context (i.e., only when participants reported that they were in the company of at least another person). The mean of the following items were used: “This company is pleasant (reversed score for analyses),” “I would rather be alone,” and “I feel judged by this company.” These items were rated on a 7-point Likert scale ranging from 1 to 7, with higher scores representing higher social stress.
**Alone stress**	Alone stress was based on the appraisal of current aloneness (i.e., only when participants reported that they were alone). The mean of the following items were used: “I enjoy being alone (reversed score for analyses),” “I feel alone,” and “I would rather be in the company of someone.” These items were rated on a 7-point Likert scale ranging from 1 to 7, with higher scores representing higher alone stress.
**Activity-related stress**	Activity-related stress was based on the average score of 2 items. “Think of the activity you were doing before the beep” (1) “I like doing this activity (reversed score for analyses)” and (2) “This activity is difficult for me.” These items were rated on a 7-point Likert scale ranging from 1 to 7. Mean score of the 2 items was taken to compute the activity-related stress value, with higher scores representing higher activity-related stress.
**Event-related stress**	Event-related stress was based on the item. “Think of the most important event that happened since the last beep: this event was pleasant.” This item was initially rated on a 7-point Likert scale ranging from −3 (very unpleasant) to 3 (very pleasant) and was transformed to a 4-point Likert scale ranging from 0 (pleasant to neutral events) to 3 (very unpleasant) for the analyses. Higher scores were thus representing higher event-related stress.

### Statistical analysis

Statistical analyses were conducted in STATA version 12.1 (StataCorp). For all analyses, the level of statistical significance was set to *p* < 0.05. Chi-square tests and analyses of variance (ANOVA) were used to investigate group differences in demographic characteristics.

Regarding the analysis of ESM data, group comparisons for time-invariant variables (i.e., one observation per participant, such as the percentage of time spent alone) were performed using multiple linear regression models, controlling for age and gender. Multilevel regression analyses were performed to compute group differences on time-varying variables (i.e., one observation per beep for each participant, which requires the use of multilevel analyses, such as positive or negative affect) using the XTMIXED command, again controlling for age and gender.

To test group differences in affective reactivity to daily-life stressors, a multilevel model was estimated using negative affect or psychotic symptoms as the dependent variable and stress (social stress, alone stress, activity-related stress, or event-related stress) and group (22q11DS vs. HCs) as the independent variables. To investigate possible differences in stress reactivity between the groups, the group×stress interaction term was also added to the model. The models correct for autocorrelation between residuals (using an AR1 autocorrelation structure) to account for autoregressive effects (observations from 1 subject that are closer to each other in time will be more similar than those further apart). The B’s represent the fixed (unstandardized) regression coefficients of the predictors in the multilevel model.

## Results

### Sample characteristics and behavioral assessments

A total of 55 participants (*n* = 31 22q11DS and *n* = 24 HC) were included in the study and completed a total of 2292 ESM reports. Four participants with 22q11DS (with a combined number of 45 valid ESM reports) had to be excluded because they did not provide enough ESM assessments (less than 33.3% of total number of beeps = 20). This resulted in a dataset of 2236 valid ESM reports from 51 subjects including *n* = 27 22q11DS who completed 1122 (68.21% compliance) ESM reports and *n* = 24 HCs who completed 1114 ESM reports (77.92% compliance). 22q11DS participants completed significantly less momentary assessments compared to HCs (Table 2). Demographics of included participants are shown in Table 2. Groups did not differ on most demographic characteristics. There were significant group differences in income situation, level of education, and medication use (Table 2). As expected, given that impaired cognitive functioning is a core characteristic of individuals with 22q11DS [36], IQ was also significantly lower in 22q11DS compared to the HC group (*F* (1, 51) = 107.73, *p* < 0.001, Table 2). It should be noted that the average IQ in the 22q11DS group was 78 (range 59‑103), which is slightly above the average IQ reported in 22q11DS (e.g., [36]), and only 4 (14.8%) participants had an IQ falling into the intellectual disability range (IQ < 70). This reflects that participants with 22q11DS included in the current study were relatively high functioning from a cognitive point of view. Of note, the number of completed beeps was not significantly associated with IQ in the 22q11 group (*r*_s_ = −0.141, *p* = 0.482).

**Table 2:** Demographic characteristics and descriptive statistics

	HC (***n*** = 24)	22q11DS (***n*** = 27)	Test statistic	***Ρ*** value
**Gender (*n*** **male:*n*** **female)**	7:17	9:18	*X*^2^(1) = 0.10	0.75
**Age in years, mean (S.D.)**	39.91 (± 13.41)	34.11 (± 9.81)	*F* = 2.16	0.15
**IQ, mean (S.D.)**	106.09 (± 8.36)	78.29 (± 10.43)	*F* = 107.73	< 0.001**
**Level of education,** ***n*** **(%)**			*X*^2^(2) = 24.22	< 0.001**
Secondary school or less^a^	1 (4.17%)	14 (51.85%)		
Further education (MBO)	8 (33.33%)	12 (44.44%)		
Higher education (HBO/WO)	15 (62.50%)	1 (3.70%)		
**Marital status,** ***n*** **(%)**			*X*^2^(1) = 1.2	0.28
Married or living together	8 (33.33%)	13 (48.15%)		
Never married/single/divorced	16 (66.67%)	14 (51.85%)		
**Living situation,** ***n*** **(%)**			*X*^2^(3) = 3.4	0.33
Alone	6 (25.00%)	4 (14.81%)		
With parents/relatives	11 (45.83%)	13 (48.15%)		
With partner/family/children/alone with children	7 (29.17%)	7 (25.93%)		
Special housing (psychiatric/non-psychiatric institute)	0 (0%)	3 (11.11%)		
**Income,** ***n*** **(%)**			*X*^2^(2) = 6.7	0.03*
Salary (work)/student fee	18 (75.00%)	11 (40.74%)		
Income from social workplace	0 (0%)	2 (7.41%)		
Income from benefit or maintenance^b^	6 (25.00%)	14 (51.85%)		
**Work situation,** ***n*** **(%)**			*X*^2^(1) = 3.8	0.05
Working/significant housework/studying	18 (75.00%)	13 (48.15%)		
Disabled or unemployed	6 (25.00%)	14 (51.85%)		
**ESM, mean (S.D.)**				
Number of beeps filled out per participant	46.79 (± 7.92)	41.62 (± 8.73)	*F* = 4.84	0.03*
Negative affect	1.50 (± 0.42)	2.04 (± 0.95)	*Z* = 2.74	0.006*
Positive affect	4.80 (± 0.74)	4.82 (± 0.99)	*Z* = 0.19	0.852
Psychotic experiences	1.19 (± 0.43)	1.69 (± 1.06)	*Z* = 2.49	0.013*
% Time alone	44.46 (± 25.26)	32.78 (± 21.61)	*T* = −14.11	< 0.001**
% Time with familiar persons	48.96 (± 24.92)	65.95 (± 22.53)	*T* = 18.86	< 0.001**
% Time with strangers	6.57 (± 9.55)	1.27 (± 3.24)	*T* = −16.59	< 0.001**
% School/work activity	26.06 (± 16.04)	11.99 (± 11.96)	*T* = −3.80	< 0.001**
% House-related activity	11.24 (± 7.67)	12.14 (± 10.43)	*T* = 0.35	0.731
% Social activity	13.81 (± 6.71)	13.54 (± 12.23)	*T* = −0.10	0.923
% Leisure activity	27.66 (± 11.88)	27.11 (± 17.27)	*T* = −0.13	0.896
% Eat/drink activity	8.05 (± 6.59)	17.10 (± 8.33)	*T* = 4.26	< 0.001**
Social stress	2.11 (± 0.65)	2.04 (± 0.85)	*Z* = 0.16	0.869
Alone stress	2.61 (± 0.79)	2.91 (± 1.00)	*Z* = 1.16	0.245
Activity-related stress	2.68 (± 0.69)	2.60 (± 1.03)	*Z* = −0.15	0.877
Event-related stress	0.07 (± 0.07)	0.17 (± 0.26)	*Z* = 1.99	0.047
**Diagnosis (M.I.N.I.),** ***n*** **(%)**				
Psychotic disorder	0 (0%)	1 (3.7%)		
Mood (and anxiety) disorder	0 (0%)	4 (14.8%)		
Only anxiety disorder	0 (0%)	3 (11.11%)		
None	24 (100%)	19 (70.4%)		
**Psychoactive medication,** ***n*** **(%)**	0 (0%)	8 (30%)^c^	*X*^2^(1) = 0.99	< 0.001**
Antipsychotics	0 (0%)	2 (7.4%)		
Antidepressants	0 (0%)	4 (14.8%)		
Mood stabilizers	0 (0%)	1 (3.7%)		
Anxiolytics	0 (0%)	1 (3.7%)		
Psychostimulants/ADHD medication	0 (0%)	2 (7.4%)		

*p < 0.05

**p < 0.001

^a^Elementary school, VMBO, LBO, HAVO, or VWO

^b^Income from benefit or maintenance due to sickness or unemployment

^c^Number of participants under psychoactive medication; can include participants with > 1 medication (*n* = 2)

### Group differences in PA, NA, and momentary psychotic experiences

Participants with 22q11DS did not significantly differ from HCs regarding the mean PA level (*b* = 0.04 (95% CI −0.43 to 0.52), *p* = 0.852). However, they reported significantly higher levels of NA compared to the control group (*b* = 0.58 (95% CI 0.16 to 0.99), *p* = 0.006) (Table 2).

Participants with 22q11DS also reported significantly higher mean levels of momentary psychotic experiences compared to HCs (*b* = 0.58 (95% CI 0.13 to 1.05), *p* = 0.013).

### Group differences in activity- and event-related stress

Participants with 22q11DS did not differ from HCs regarding the overall reported level of activity-related stress (*b* = −0.04 (95% CI −0.52 to 0.44), *p* = 0.877). In order to explore if the lack of group differences in activity-related stress could be influenced by an involvement in different types of activities, a post hoc comparison between the two groups was performed on the type of activity (Table 2). This revealed that participants with 22q11DS were less frequently involved in school/work-related activity (*b* = −14.96 (95% CI −22.87 to −7.06), *p* < 0.001) and more frequently reported to be eating/drinking (*b* = 9.04 (95% CI 4.77 to 13.31), *p* < 0.001) than HCs.

Participants with 22q11DS reported higher levels of event-related stress compared to HCs (*b* = 0.11 (95% CI 0.001 to 0.22), *p* = 0.047). To better understand this difference in the level of event-related stress, we examined the answers of the participants from both groups. Interestingly, the presence of minimally unpleasant events (rating = 1) was reported by HCs in most instances (mean percentage of unpleasant events rated as “1” = 80.2%) compared to the 22q11DS group (mean percentage of unpleasant events rated as “1” = 45.3%; *t* = −20.21, *p* < 0.001). Conversely, the presence of extremely unpleasant events (rating = 3) was never reported in the HC group (mean percentage of unpleasant events rated as “3” = 0%) but was frequently reported among participants with 22q11DS (mean percentage of unpleasant events rated as “3” = 35.3%; *t* = 28.65, *p* < 0.001). The presence of moderately unpleasant events (rating = 2) did not differ between the two groups (mean percentage of unpleasant events rated as “2” in HC = 19.8%; mean percentage of unpleasant events rated as “2” in 22q11DS = 19.5%; *t* = 0.81, *p* = 0.419).

### Group differences in alone and social stress

Overall, alone stress (*b* = 0.31 (95% CI −0.21 to 0.83), *p* = 0.245) and social stress (*b* = 0.04 (95% CI −0.39 to 0.46), *p* = 0.869) were not significantly different between the two groups (Table 2). Both groups of participants reported higher levels of social stress when in the company of strangers compared to familiar persons (*b* = 0.78 (95% CI 0.38 to 1.18), *p* < 0.001). However, the interaction effect between group and familiarity status was not significant (*b* = 0.17 (95% CI −0.56 to 0.89), *p* = 0.654), indicating that both participants with 22q11DS and HCs had a similar increase of social stress when in the company of strangers.

In order to explore if the lack of group difference in alone stress and social stress could be influenced by a different involvement in the social world, a post hoc comparison between the two groups was performed on the percentage of time spent alone, in the company of familiar persons and in the company of strangers. This comparison revealed that participants with 22q11DS spent significantly less time alone and less time with strangers compared to HCs but significantly more time in the company of familiar persons (Table 2).

### Group differences in affective and psychotic reactivity to daily-life stressors

In both groups of participants, an increase in social stress was significantly associated with an increase in NA (*b* = 0.21 (95% CI 0.09 to 0.34), *p* = 0.001). A similar association was observed between alone stress and NA (*b* = 0.15 (95% CI 0.06 to 0.25), *p* = 0.002), between activity-related stress and NA (*b* = 0.15 (95% CI 0.08 to 0.21), *p* < 0.001), and between event-related stress and NA (*b* = 0.39 (95% CI 0.18 to 0.60 *p* < 0.001). However, the group×stress interaction on NA was not significant for any of the stress variables (for social stress, *b* = 0.02 (95% CI −0.16 to 0.19), *p* = 0.859; for alone stress, *b* = 0.07 (95% CI −0.08 to 0.21), *p* = 0.362; for activity-related stress, *b* = 0.03 (95% CI −0.06 to 0.12), *p* = 0.549); for event-related stress, *b* = −0.03 (95% CI −0.31 to 0.24), *p* = 0.816), indicating similar affective reactivity to stress in both groups of participants.

For the entire group, the level of momentary psychotic experiences was not significantly associated with any of the stress variables (for social stress, *b* = 0.03 (95% CI −0.05 to 0.13), *p* = 0.400; for alone stress, *b* = 0.03 (95% CI −0.07 to 0.14), *p* = 0.517; for activity-related stress, *b* = 0.03 (95% CI −0.03 to 0.78), *p* = 0.335; for event-related stress, *b* = −0.06 (95% CI −0.11 to 0.22), *p* = 0.486). The group×stress interaction on psychotic experiences was also not significant for any of the stress variables (for social stress, *b* = 0.01 (95% CI −0.12 to 0.14), *p* = 0.884; for alone stress, *b* = 0.002 (95% CI −0.15 to 0.15), *p* = 0.982; for activity-related stress, *b* = 0.01 (95% CI −0.06 to 0.09), *p* = 0.712; for event-related stress, *b* = 0.03 (95% CI −0.18 to 0.25), *p* = 0.752).

## Discussion

This is the first study to investigate affective states, perceived stress, and affective and psychotic reactivity to various sources of environmental stress in 22q11DS using ESM. Our main findings indicate that adults with 22q11DS display overall higher NA and momentary psychotic experiences throughout the day and report a higher level of event-related stress. On the other hand, participants with 22q11DS reported (1) similar levels of PA throughout the day, (2) similar levels of social stress, alone stress, and activity-related stress, (3) a similar increase of social stress when in the company of strangers compared to familiar persons, and (4) a comparable affective and psychotic reactivity in relation to all types of environmental stress (social stress, alone stress, activity-related stress, and event-related stress) compared to HCs.

### Affective states and perceived stress in daily life

Firstly, we observed that adults with 22q11DS reported higher levels of NA throughout the day compared to healthy controls. This finding might be related to the fact that participants with 22q11DS also reported a higher level of event-related stress compared to HCs (i.e., experience of an unpleasant event since the last beep). In particular, this result was mainly driven by the fact that participants with 22q11DS often reported the presence of extremely unpleasant events, whereas HCs mainly reported the presence of minimally unpleasant events in the flow of daily life. Interestingly, two recent studies reported reduced exposure to stressful life events [23], as measured with the Coddington Life Event Scale (CLES), and comparable exposure to traumatic events [28], as measured with the Childhood Trauma Questionnaire (CTQ), in individuals with 22q11DS compared to HCs, which might appear in contradiction with the present findings. However, whereas the CLES or CTQ target discrete life events that happen at a relatively low frequency (e.g., changing school/work or being the victim of violence), the current study might rather reflect an increased prevalence of minor events that are subjectively experienced as stressful by individuals with 22q11DS, such as ongoing daily hassles (i.e., demands or conditions of daily living that have been appraised as salient and harmful or threatening to the endorser’s well-being; [37]). A qualitative study should examine the type of events that are appraised as subjectively stressful in this population to help targeting specific stress management programs.

Increased NA and the tendency to experience daily-life events as subjectively more stressful in 22q11DS could be related to a biological vulnerability inherent to this population. Indeed, several genes included in the 22q11.2 region have been involved in the biological response to stress. For example, this is the case for the catechol O-methyltransferase (COMT) gene that encodes the enzyme responsible for breaking down catecholamines, including noradrenaline and extracellular dopamine. In particular, its functional polymorphism at position 158, resulting in a valine (Val) to methionine (Met) substitution is associated with a 30% reduction in enzymatic activity [38] and has been shown to alter the HPA-axis functioning, cortisol levels, subjective feelings of stress and stress reactivity [39, 40]. For example, van Winkel et al. [40] observed that individuals with schizophrenia carrying the high-activity COMT allele experienced greater affective and psychotic reactivity to stress in daily life compared to those carrying the low-activity COMT allele. Another potential candidate is the proline dehydrogenase (PRODH) gene that regulates glutamate and γ-aminobutyric acid (GABA). For example, PRODH variant has been linked to elevated prepulse inhibition and greater anxiety in adults from the general population [41], and elevated proline level has been associated with impaired brain function in 22q11DS [42].

Contrary to our expectations, we did not observe any significant difference between individuals with 22q11DS and HCs in the intensity of perceived social stress (i.e., negative appraisals of the current social company), as well as a similar increase of social stress in the two groups when in the company of strangers compared to familiar persons. This appears in contrast with previous findings reporting a relatively high prevalence of social anxiety and social phobia in this population (e.g., [3]). It should be noted that participants with 22q11DS spent more time in the company of familiar persons and less time with strangers compared to HCs, suggesting a qualitatively different involvement in the social world in this population. The increased frequency of contacts with familiar persons could be due to an increased willingness to seek familiar company, or a lack of opportunity to be in the company of less familiar persons. Indeed, a higher proportion of adults in the 22q11DS group were unemployed at the time of the study, which may limit the opportunities to be in the company of individuals who are not part of their intimate social circle or institutional environment. Regardless of the reasons underlying this different involvement in the social world, the fact that individuals with 22q11DS rarely reported to be in the presence of strangers (1.27% on average) might have prevented a reliable estimation of social stress in this specific context. Of note, the majority of previous research focusing on social anxiety has been conducted on children and adolescent samples, which limits direct comparison with the results of the present study. Future ESM studies should also involve younger individuals with 22q11DS to explore the presence of potential developmental effects in the appraisal of social interactions and social stress.

The two groups of participants also did not significantly differ in the intensity of perceived activity-related stress (i.e., appraisal of being involved in an unpleasant and difficult activity). This lack of difference in activity-related stress might be due to a differential involvement in various types of activities between the two groups. In particular, participants with 22q11DS were less often engaged in school or work-related activities and reported more often to be eating or drinking. This difference in the activity profile could suggest that individuals with 22q11DS are less often involved in activities that are likely to be appraised as unpleasant and/or difficult.

### Affective and psychotic reactivity to daily-life stress

Contrary to our hypothesis, we did not observe an atypical affective or psychotic reactivity to stress (i.e., increase of NA or psychotic experiences following stress) in individuals with 22q11DS compared to HCs. This lack of significant difference, at least regarding affective reactivity to stress, might be driven by the overall high NA level in 22q11DS group. Indeed, at least a subgroup of individuals with 22q11DS might experience pervasive NA in their daily life, regardless of exposure to stressful experiences, which may limit the possibility to observe a large increase in NA following a stress (i.e., this would be comparable to a ceiling effect problem). Alternatively, the lack of a significant group difference in reactivity to stress could also be driven by the fact that only adults were included in the present study. Indeed, previous studies have shown that adults with 22q11DS are characterized by a reduced volume of the pituitary gland, low mean salivary cortisol, and a blunted cortisol reactivity to stress compared to HCs [23, 28], whereas increased salivary cortisol and a similar volume of the pituitary gland than HCs were reported in younger individuals [23, 43, 44]. As suggested by van Duin et al. [28], differences across age groups might be explained by chronic overactivation of the HPA-axis, eventually resulting in an “exhaustion” of the cortisol response to stress, similar to what has been described in posttraumatic stress disorder (PTSD) (e.g., [45]). Following this hypothesis, one could expect increased affective and psychotic reactivity to daily-life stress in children and adolescents with 22q11DS that then decreases by the time individuals reach adulthood. This of course remains speculative at this stage and future ESM studies should be conducted in this population on a broader age range to specifically investigate the presence of potential developmental effects.

### Strengths, limitations, and future directions

This is the first study investigating affective and psychotic reactivity to daily-life experiences in a group of adults with 22q11DS. ESM is a well-established method to investigate daily affective fluctuations, while taking contextual information into account. Furthermore, 22q11DS is a well-defined genetic syndrome exhibiting multisystem problems and conferring increased risk for developing psychiatric disorders, which makes the findings of our study interesting for the broader investigation of mediating factors of psychiatric disorders.

The present study should be viewed in light of some methodological considerations. First of all, although the ESM method allows for high ecological validity assessment of real-world experiences and activities, it relies on subjective self-report by the participant. The interpretation of questions may therefore differ between individuals and groups. In particular, it should be highlighted that the content of the ESM protocol has not been previously validated in the 22q11DS population.

Second, although ESM has been validated in multiple studies, to our knowledge it has rarely been used in the 22q11DS population (but see [28]) and more generally in individuals with neurodevelopmental disorders (but see [46]). Even if the number of participants who had to be excluded from the final analyses (because they answered to less than a third of the emitted beeps) was acceptable, the total number of completed beeps was lower in the 22q11DS group but was not significantly associated with IQ. Of note, a recent study by Wilson et al. recently demonstrated the feasibility of the ESM technique in adults with mild to moderate intellectual disability [46]. To account for the possible effect of the cognitive impairments often seen in 22q11DS, we took extra care in explaining and practicing the PsyMateTM protocol. Our personal experience was also that a subgroup of participants required additional monitoring during the ESM period, which highlights the importance of having a member of the research team dedicated to the project who is easily reachable by SMS, phone, or e-mail in case the participants have questions or experience technical issues. Methodological studies examining in greater detail the validity of ESM and compliance with this technique (and the predictors thereof) in individuals with neurodevelopmental disorder should be conducted in the future studies. It would also be important to assess the impact of changes to the ESM assessment protocol, as described in the article by Wilson et al. [46] (e.g., use of pictograms along with the text; dichotomous responses instead of responses on a Likert scale), on compliance. Also, it cannot be excluded that participants (especially in the 22q11DS given the lower response rate than in HCs) chose to avoid answering to an ESM beep in specific situations, which might introduce a response bias.

Furthermore, although the current ESM items have been previously used in studies conducted in various clinical groups, the different items measuring stress as well as momentary psychotic experiences have never been investigated in the context of a methodological study in order to examine their psychometric properties. This should be done to potentially optimize these items in future research.

Finally, it should be considered when interpreting the results that the 22q11DS sample was heterogeneous, with several individuals using psychoactive medication and being diagnosed with one or more psychiatric diagnoses. However, the majority consisted of relatively well-functioning adults with, on average, slightly higher cognitive levels than what is typically reported in 22q11DS. Therefore, the conclusions of the present study should not be extrapolated to the entire 22q11DS population.

## Conclusions

The results of the present ESM study indicate that adults with 22q11DS experience more negative affect throughout the day and increased momentary psychotic experiences than HCs. They also reported a subjectively higher level of stress when exposed to daily-life events compared to the control group, suggesting a more negative appraisal of ongoing daily hassles. However, both groups were comparable in terms of affective and psychotic reactivity to daily-life stress. Altogether, previous literature and the results of this study highlight the need to better characterize reactivity to daily-life stressors in this population, such as ongoing daily hassles, in a developmental perspective.

## Data Availability

The datasets used and analyzed for the current study are available from the corresponding author on reasonable request.

## Abbreviations

22q11DS: 22q11.2 deletion syndrome
ANOVA: Analysis of variance
CIDI: Composite International Diagnostic Interview
CLES: Coddington Life Event Scale
COMT: Catechol O-methyltransferase
CTQ: Childhood Trauma Questionnaire
ESM: Experience sampling method
FISH: Fluorescence in situ hybridization
GABA: Glutamate and γ-aminobutyric acid
HC: Healthy controls
HPA axis: Hypothalamic-pituitary-adrenocortical axis
Met: Methionine
M.I.N.I.: Mini-international neuropsychiatric interview
MLPA: Multiplex ligation-dependent probe amplification
NA: Negative affect
PA: Positive affect
PRODH: Proline dehydrogenase
PTSD: Posttraumatic stress disorder
Val: Valine
WAIS: Wechsler Adult Intelligence Scale
